# Emodin, a Naturally Occurring Anthraquinone Derivative, Ameliorates Dyslipidemia by Activating AMP-Activated Protein Kinase in High-Fat-Diet-Fed Rats

**DOI:** 10.1155/2012/781812

**Published:** 2012-05-10

**Authors:** Thing-Fong Tzeng, Hung-Jen Lu, Shorong-Shii Liou, Chia Ju Chang, I-Min Liu

**Affiliations:** ^1^Department of Internal Medicine, Pao Chien Hospital, Pingtung City, Pingtung County 90064, Taiwan; ^2^Traditional Medicinal Center, Kaohsiung Veterans General Hospital, Kaohsiung City 81362, Taiwan; ^3^Department of Pharmacy and Graduate Institute of Pharmaceutical Technology, Tajen University, Yanpu Shiang, Ping Tung Shien 90701, Taiwan; ^4^School of Chinese Pharmaceutical Sciences and Chinese Medicine Resources, China Medical University, Taichung 40402, Taiwan

## Abstract

The aim of this study was to investigate the antiobesity and antihyperlipidaemic effects of emodin on high-fat diet (HFD)-induced obese rats, and on the regulation of the expression of the genes involved in lipid metabolism to elucidate the mechanisms. After being fed HFD for two weeks, Wistar rats were dosed orally with emodin (40 and 80 mg kg^−1^) or pioglitazone (20 mg kg^−1^), once daily for eight weeks. Emodin (80 mg kg^−1^ per day) displayed similar characteristics to pioglitazone (20 mg kg^−1^ per day) in reducing body weight gain, plasma lipid levels as well as coronary artery risk index and atherogenic index of HFD-fed rats. Emodin also caused dose related reductions in the hepatic triglyceride and cholesterol contents and lowered hepatic lipid droplets accumulation in HFD-fed rats. Emodin and pioglitazone enhanced the phosphorylation of AMP-activated protein kinase (AMPK) and its primary downstream targeting enzyme, acetyl-CoA carboxylase, up-regulated gene expression of carnitine palmitoyl transferase 1, and down-regulated sterol regulatory element binding protein 1 and fatty acid synthase protein levels in hepatocytes of HFD-fed rats. Our findings suggest emodin could attenuate lipid accumulation by decreasing lipogenesis and increasing mitochondrial fatty acid **β**-oxidation mediated by activation of the AMPK signaling pathway.

## 1. Introduction

Obesity accelerates the accumulation of excessive fat, which can cause chronic diseases in both humans and laboratory animals such as diabetes mellitus, cardiovascular disease, digestive disease, respiratory disease, and various cancers [1]. To treat and control obesity, besides diet therapy and exercise, many different approaches such as drugs for weight loss or loss of appetite and food supplements are suggested to date. However, most antiobesity drugs have been withdrawn from the market based on U.S. FDA warnings of serious adverse reactions [2]. There is growing interest in herbal remedies due to the side effects associated with current antiobesity agents, because the plant kingdom is a wide field to search for natural effective antiobesity agents with less significant side effects. In line with this, we used oriental medicinal herbs that had been expected to have a lipid lowering effect based on the previous reports [3].

Emodin (1,3,8-trihydroxy-6-methylanthraquinone), an important component of rhubarb, a traditional Chinese herb, exerts an obvious antiinflammatory [4, 5] and anti-oxidative effects [6, 7]. Emodin could prevent the formation and progress of atherosclerosis by inhibiting the proliferation of human vascular smooth muscle cells and reducing the plasma concentration of malondialdehyde and oxidized low density lipoprotein [8]. That emodin ameliorated the glucose tolerance and improved insulin sensitivity through the activation of peroxisome-proliferator-activated receptor (PPAR)*γ* similar to thiazolidinediones (TZD) on diabetic mice has been proved [9]. Actually, the fact that TZD activates AMP-activated protein kinase (AMPK) to stimulate the pathways that increase energy production, such as glucose transport and fatty acid oxidation, and switches off the pathways that consume energy such as lipogenesis, protein have been identified [10]. Although emodin has also been found to be effective in fatty liver associated with increasing the expression of hepatic PPAR*γ* [11], insufficient information is available regarding the mechanism related to AMPK signaling pathway on the regulation of lipid disorders.

It is well known that high-fat diets (HFDs) are responsible for high global prevalence of obesity [12]. Rodents fed a lard-based HFD showed visceral adiposity, hyperglycemia, dyslipidemia, hyperinsulinemia, and hepatic steatosis, which are distinctly linked with human obesity [13]. This study investigated the effects of emodin on lipid metabolism in HFD-fed rats and sought possible mechanisms of action.

## 2. Methods

### 2.1. Animal Models and Treatment Protocols

Male Wistar rats aged 8 weeks were obtained from the National Laboratory Animal Center (Taipei, Taiwan). They were maintained in a temperature-controlled room (25 ± 1°C) and kept on a 12:12 light-dark cycle (lights on at 06:00 h) in our animal center. Food and water were available *ad libitum*. Regular rat diet (RCD) (11 kcal% fat #LM-485, Teklad, Madison, WI) was used as the maintenance and control diet. A purified ingredient HFD with 45 kcal% fat primarily from lard (no. D12451, Research Diets, New Brunswick, NJ) was used to induce a rapid increase in body weight (BW) and obesity [14]. The caloric density of the control diet was 3.4 kcal g^−1^; that of the HFD was 4.73 kcal g^−1^, resulting in lower daily food consumption in grams for the rats fed the HFD. All animal procedures were performed according to the Guide for the Care and Use of Laboratory Animals of the National Institutes of Health, as well as the guidelines of the Animal Welfare Act (approval number: IACUC 99-16; approval date: September 9, 2010).

After being fed an HFD for 2 weeks, emodin (purity ≥97.0%; Sigma-Aldrich Co.; Cat. no. 30269) was dissolved in distilled water for oral gavage administration at the desired doses (40 and 80 mg kg^−1^ per day) in a volume of 2 mL kg^−1^ once a day into the HFD-fed rats. The dose of emodin chosen was according to previous study indicated that emodin at an oral dose of 40 mg kg^−1^ was effective in the murine nonalcoholic fatty liver (NAFLD) treatment [11]. Another group of HFD-fed was treated by oral gavage with pioglitazone hydrochloride (Takeda Pharmaceutical Co. Ltd. Osaka, Japan: 20 mg kg^−1^ per day) [15]. A further group of HFD-fed and RCD-fed rats were treated similarly but with the same volume of vehicle (distilled water) as was used to dissolve the tested compounds during the same treatment period. Water was made available *ad libitum* throughout the experiment.

Eight weeks after the treatment, rats were weighed and blood samples were collected from the lateral tail vein of the animals anesthetized with sodium pentobarbital (30 mg kg^−1^) administered intraperitoneally (i.p.). Samples were centrifuged at 2,000 ×*g * for 10 minutes at 4°C. The plasma was then removed and placed into aliquots for the respective analytical determinations. Livers were removed after blood was collected, rinsed with a physiological saline solution, weighed, and immediately stored at −70°C.

### 2.2. Biochemical Parameter Analysis

 The diagnostic kits for determinations for plasma glucose (Cat. no. 10009582), total cholesterol (TC; Cat. no. 10007640) and triglyceride (TG; Cat. no. 10010303) were purchased from Cayman Chemical Company (Michigan, USA). The diagnostic kit for determinations for plasma levels of high -density lipoprotein cholesterol (HDL-c) was purchased from Bio-Quant Diagnostics (CA, USA; Cat. No. BQ 019CR), low-density lipoprotein cholesterol (LDL-c) were calculated by using Friedewald's equation [16]. Plasma level of free fatty acids (FFAs) was determined by the FFAs quantification kit obtained from Abcam plc. (MA, USA; Cat. No. ab65341). All samples were analyzed in triplicate. Atherogenic index (AI) and coronary risk index (CRI) were calculated as: LDL-c/HDL-c and TC/HDL-c [17, 18], respectively.

### 2.3. Extraction of Hepatic Lipids

 After removal from the animals, part of the samples of fresh liver were collected for analyzing the lipid contained. Liver (1.25 g) was homogenized with chloroform/methanol (1 : 2, 3.75 mL), and then chloroform (1.25 mL) and distilled water (1.25 mL) were added to the homogenate and mixed well. After centrifugation (1,500 ×*g* for 10 min), the lower clear organic phase solution was transferred into a new glass tube and then lyophilized. The lyophilized powder was dissolved in chloroform/methanol (1 : 2) as the hepatic lipid extracts and stored at −20°C for less than 3 days [19]. The hepatic cholesterol and triglyceride in the lipid extracts were analyzed with the diagnostic kits, which were used in the plasma analysis.

### 2.4. Hepatic Pathological Evaluation

 Small pieces of hepatic tissues taken from experimental animals were fixed in 10% neutral formalin, alcohol-dehydrated, paraffin-embedded, and sectioned to a mean thickness of 4 *μ*m. The histological examination by the above conventional methods was evaluated for the index of diabetic-induced necrosis by assessing the morphological changes with hematoxylin and eosin (H&E) stain. Liver biopsy was scored according to the criteria as follows: [20] grade 0, no steatosis, normal liver; grade 1, <25% of hepatocytes affected; grade 2, 26–50% of hepatocytes affected; grade 3, 51–75% of hepatocytes affected; grade 4, >76% of hepatocytes affected.

### 2.5. Preparation of Hepatic Fractions

 The hepatic tissue was homogenized with ice-cold lysis buffer (pH 7.4) containing 137 mmol L^−1^ NaCl, 20 mmol L^−1^ Tris-HCl, 1% Tween 20, 10% glycerol, 1 mmol L^−1^ phenylmethylsulfonyl fluoride (PMSF), and protease inhibitor mixture DMSO solution. The homogenate was then centrifuged at 2,000 ×*g* for 10 min at 4°C. The protein concentration of each fraction was determined using a commercial kit (Bio-Rad Laboratories, Hercules, CA, USA).

### 2.6. Western Blot Analyses

 After homogenization of hepatic tissue samples using a glass/Teflon homogenizer, protein concentrations were determined using the BioRad protein dye binding assay. The homogenates (50 *μ*g) were separated by sodium dodecyl sulfate-polyacrylamide gel electrophoresis and Western blot analysis was performed using either an anti-rat antibody to bind acetyl-CoA carboxylase (ACC) (Santa Cruz Biotechnology, Inc., CA, USA; Cat. no. 3662), phospho-ACC at Ser 79 (p-ACC; Cell Signaling Technology, MA, USA), AMPK*α* (Cell Signaling Technology; Cat. no. 2352), phospho-AMPK*α* at Thr 172 (p-AMPK*α*; Cell Signaling Technology; Cat. no. 2531), carnitine palmitoyl transferase 1 (CPT-1; Cell Signaling Technology; Cat. no. sc-98834), sterol regulatory element binding protein 1 (SREBP-1; Santa Cruz Biotechnology, Inc.; Cat. no. sc-366), fatty acid synthase (FAS; Cell Signaling Technology; Cat. no. 3180) or  *β*-actin (Santa Cruz Biotechnology, Inc.; Cat. no. sc-130656). Blots were incubated with the appropriate peroxidase-conjugated secondary antibody. The membranes were then washed three times in TBST (20 mmol L^−1^ Tris-HCl, pH 7.5), 150 mmol L^−1^ NaCl, and 0.05% Tween 20 and visualized on X-ray film using the enhanced chemiluminescence detection system (Amersham Corp., Braunschweig, Germany). Densitometric analysis of the bands for the expression of protein was done with ATTO Densitograph Software (ATTO Corporation, Tokyo, Japan).

### 2.7. Statistical Analysis

 All data represented as the mean ± SEM. Statistical differences among groups were determined by using two-way repeated-measures ANOVA. The Dunnett range post hoc comparisons were used to determine the source of significant differences where appropriate. A *P* value <.05 was considered statistically significant.

## 3. Results

### 3.1. Effects on Body Weight (BW) and Feeding Behavior

 The BW gain was significantly higher in HFD-fed rats than that in the RCD-fed group (Table 1). Eight weeks after emodin treatment, the BW gain of rats was significantly lower than that of rats in the HFD group. Emodin (80 mg kg^−1^ per day) significantly suppressed BW gain; similar results were seen in rats treated with pioglitazone (20 mg kg^−1^ per day; Table 1).

Although the water intake became slightly higher in vehicle-treated HFD-fed group as compared to the others, no significant differences were observed in daily food intake among all the groups during whole experimental period (Table 1).

### 3.2. Effect on Plasma Lipids

The HFD caused the elevation of plasma TC, TG, and LDL-c concentrations in rats (Table 2). Emodin at the daily oral dosage of 40 and 80 mg kg^−1^ significantly decreased the level of plasma TC (23.5% and 31.6% reduction, resp.) in HFD-fed rats compared to their vehicle counterparts (Table 2). All doses of emodin decreased the level of plasma TG in HFD-fed rats (Table 2). Emodin at the daily oral dosage of 40 and 80 mg kg^−1^ reduced plasma levels of LDL-c by 38.2% and 52.1%, respectively, (Table 2). Plasma TC, TG, and LDL-c concentrations were significantly lower by 38.4%, 47.3%, and 59.7%, respectively in pioglitazone- (20 mg kg^−1^ per day) treated HFD-fed rats compared with vehicle-treated counterparts (Table 2).

Plasma level of HDL-c in HFD-fed rats was lower to 59.3% of that in the RCD-fed group (Table 2). After 8 weeks of treatment with emodin (80 mg kg^−1^ per day) or pioglitazone (20 mg kg^−1^ per day), lower plasma level of HDL-c in HFD-fed rats was elevated to nearly that of RCD-fed group (Table 2).

The plasma FFAs were significantly higher in HFD-fed rats receiving vehicle (Table 2). After 8 weeks of treatment, plasma FFA was lower by 37.5% in emodin- (80 mg kg^−1^ per day) treated HFD-fed rats as compared with the vehicle-treated counterparts (Table 2). FFA concentrations in HFD-fed rats were significantly lower by 46.3% in 8-weeks pioglitazone-treated, HFD-fed rats compared to their vehicle counterparts (Table 2).

Pioglitazone treatment arrested the elevation of AI and CRI in HFD-fed rats (Table 2). Emodin administration also caused a dose-related reduction in the atherogenic and coronary artery risk indices in the HFD-fed rats when compared to the values recorded for the vehicle-treated counterparts (Table 2).

### 3.3. Effect of Treatment on Hepatic Lipids

**Figure 1:**
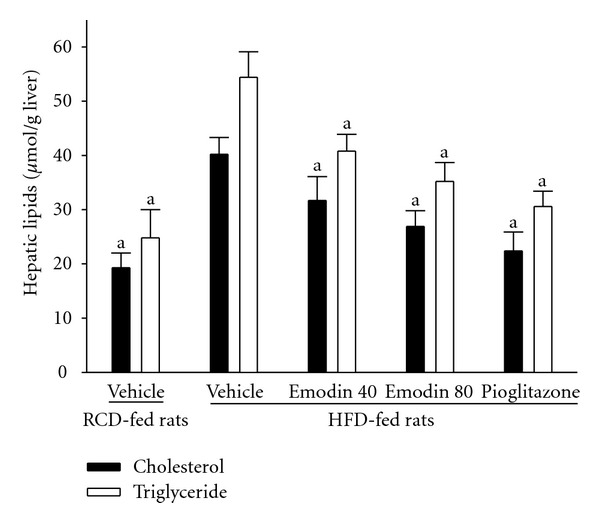
Changes in the hepatic cholesterol and triglyceride in HFD-fed rats receiving 8-week treatment. Rats not receiving any treatment were given the same volume of vehicle (distilled water) used to dissolve the test medications. Hepatic lipids were analyzed from vehicle-treated RCD-fed rats, vehicle-treated HFD-fed rats, emodin- (40 mg kg^−1^ per day) treated HFD-fed rats- (emodin 40), emodin- (80 mg kg^−1^ per day) treated HFD-fed rats (emodin 80) or pioglitazone (20 mg kg^−1^ per day)-treated HFD-fed rats (pioglitazone). Values (mean ± SEM) were obtained from each group of 8 animals in each group after 8 weeks of the experimental period. ^a^ *P* < .05 compared to the values of vehicle-treated HFD-fed rats in each group, respectively.

 The hepatic TC level was significantly higher in HFD-fed rats than that in the RCD-fed group, those were lower by 35% in the 8-week emodin- (80 mg kg^−1^ per day) treated group (Figure 1). HFD-fed rats receiving 8-week treatment with emodin- (80 mg kg^−1^ per day) also showed significantly lower values of hepatic TG to 64.7% as compared with the vehicle-treated counterparts (Figure 1). Hepatic TC and TG levels in HFD-fed rats were significantly lower by 44.3% and 43.8%, respectively, in pioglitazone-treated rats compared to their vehicle-counterparts (Figure 1).

### 3.4. Morphological Changes in Hepatocytes

**Figure 2:**
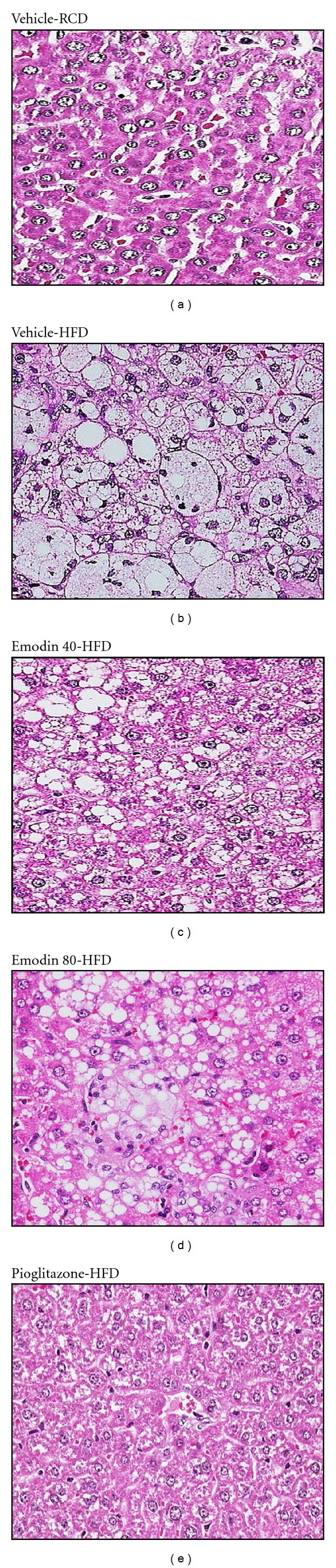
Histopathological findings in livers of HFD-fed rats receiving 8-week treatment. Rats not receiving any treatment were given the same volume of vehicle (distilled water) used to dissolve the test medications. Photomicrographs are of tissues isolated from vehicle-treated RCD-fed rats (vehicle-RCD), vehicle-treated HFD-fed rats (vehicle-HFD), emodin- (40 mg kg^−1^ per day) treated HFD-fed rats- (emodin 40-HFD), emodin (80 mg kg^−1^ per day) treated HFD-fed rats (emodin 80-HFD) or pioglitazone- (20 mg kg^−1^ per day) treated HFD-fed rats (pioglitazone-HFD). Photomicrographs were taken at a magnification of x400.

 HFD-fed rats showed considerable hepatic lipid accumulation compared with that in RCD-fed group (Figure 2). After 8 weeks, the extent of hepatic lipid accumulation in pioglitazone-treated HFD-fed rats was similar to those in RCD-fed rats (Figure 2). The pathological grading of hepatic steatosis in HFD-fed rats was 3.1 ± 0.6, which was decreased to 1.7 ± 0.5 in pioglitazone-treated group. HFD-fed rats receiving 8-week treatment with emodin showed considerably lower hepatic lipid accumulation than in their vehicle-treated counterparts (Figure 2). The pathological grading of hepatic steatosis in HFD-fed rats was reduced to 2.7 ± 0.4 and 2.0 ± 0.8 after receiving an 8-week treatment with emodin at the daily dose of 40 or 80 mg kg^−1^, respectively.

### 3.5. Protein Expression and Phosphorylation of AMPK and ACC in Hepatocytes

**Figure 3:**
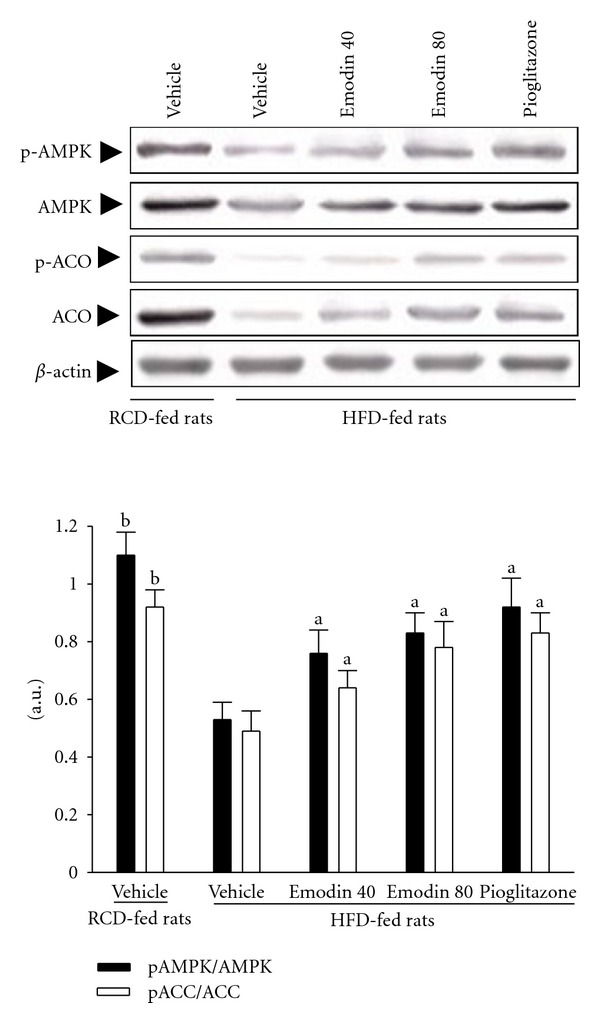
Representative immunoblots of hepatic protein levels and phosphorylation degrees of AMPK and ACC in HFD-fed rats receiving 8-week treatment. Rats not receiving any treatment were given the same volume of vehicle (distilled water) used to dissolve the test medications. Immunoblots are of tissues isolated from vehicle-treated RCD-fed rats, vehicle-treated HFD-fed rats, emodin- (40 mg kg^−1^ per day) treated HFD-fed rats (emodin 40), emodin- (80 mg kg^−1^ per day) treated HFD-fed rats (emodin 80) or pioglitazone- (20 mg kg^−1^ per day) treated HFD-fed rats (pioglitazone). Similar results were obtained with an additional 4 replications. Phospho-AMPK/AMPK and phospho-ACC/ACC protein expression were expressed as mean with SEM (*n* = 5 per group) in each column. ^a^ *P* < .05 and ^b^ *P* < .01 compared to the values of vehicle-treated HFD-fed rats.

The immunoblot results showed that the HFD led to a decrease in the protein levels and phosphorylation degree of AMPK in the hepatocytes as compared to the RCD-fed rats (Figure 3). Similarly, both the protein levels and phosphorylation degree of ACC in the hepatocytes of HFD-fed rats were downregulated than those of the RCD-fed group (Figure 3). Furthermore, the HFD significantly reduced the phospho-AMPK/AMPK ratio (by 51.8% relative to those in vehicle-treated HFD-fed rats, *P* < .05) and the phospho-ACC/ACC ratio (by 46.7% relative to those in vehicle-treated HFD-fed rats, *P* < .05) in the hepatocytes of the rats (Figure 3). These HFD-induced downregulations in the ratio of phospho-AMPK/AMPK and the phospho-ACC/ACC were significantly reversed in the hepatocytes after 8-week treatment with emodin (80 mg kg^−1^ per day) (56.6% and 59.2% increases relative to those in vehicle-treated HFD-fed rats, resp.; *P* < .05) (Figure 3). Treatment of HFD-fed rats with pioglitazone also significantly upregulated the ratio of phospho-AMPK/AMPK and the phospho-ACC/ACC in the hepatocytes to 1.7-fold and 1.6-fold relative to those in vehicle-treated HFD-fed rats (*P* < .05, Figure 3).

### 3.6. Protein Expression of SREBP-1, FAS, and CPT-1 in Hepatocytes

**Figure 4:**
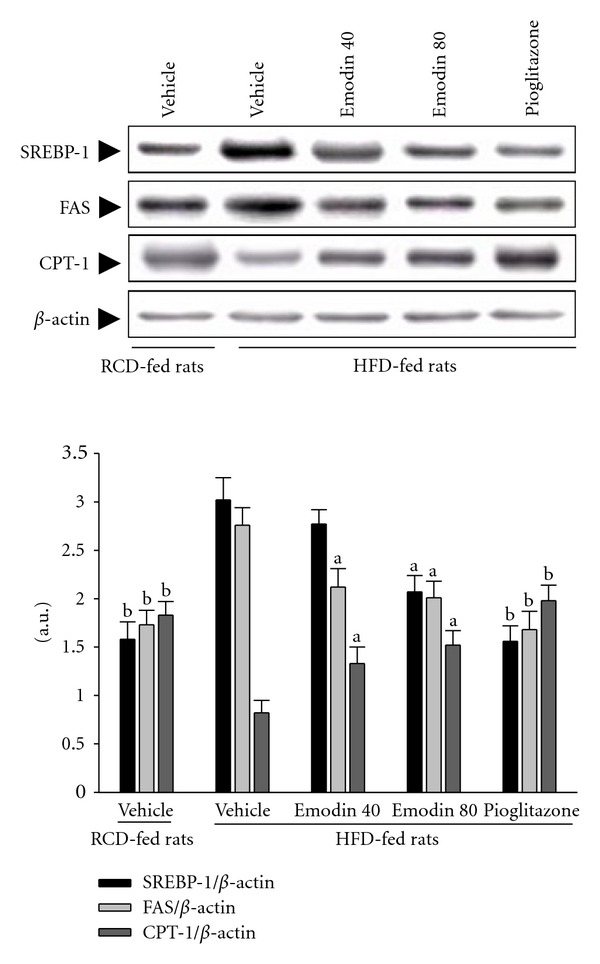
Representative immunoblots of hepatic protein levels of SREBP-1, FAS, and CPT-1 in HFD-fed rats receiving 8-week treatment. Rats not receiving any treatment were given the same volume of vehicle (distilled water) used to dissolve the test medications. Immunoblots are of tissues isolated from vehicle-treated RCD-fed rats, vehicle-treated HFD-fed rats, emodin (40 mg kg^−1^ per day)-treated HFD-fed rats (emodin 40), emodin- (80 mg kg^−1^ per day) treated HFD-fed rats (emodin 80), or pioglitazone- (20 mg kg^−1^ per day) treated HFD-fed rats (pioglitazone). Similar results were obtained with an additional 4 replications. Protein levels are expressed as the mean with SEM (*n* = 5 per group) in each column. ^a^ *P* < .05 and ^b^ *P* < .01 compared to the values of vehicle-treated HFD-fed rats in each group, respectively.

The immunoblot results showed that the protein expressions of hepatic SREBP-1 and FAS in HFD-fed rats were higher than those of RCD-fed group (Figure 4). The HFD-fed rats treated with pioglitazone for 8 weeks had hepatic SREBP-1 protein expression levels that were 48.3% lower than those in vehicle-treated counterpart (*P* < .05, Figure 4). Hepatic SREBP-1 protein expression levels in HFD-fed rats after 8-week treatment with emodin (80 mg kg^−1^ per day) were decreased by 31.6% relative to expression levels in vehicle-treated HFD-fed rats (*P* < .05, Figure 4). The HFD-induced increase in the hepatic FAS protein expression was also reversed by feeding the rats with the emodin (80 mg kg^−1^ per day) and pioglitazone (28.1% and 39.1% decreases relative to those in vehicle-treated HFD-fed rats, respectively; *P* < .05).

The immunoblot results showed that the hepatic protein levels of CPT-1 in HFD-fed rats were lower than those of RCD-fed group (*P* < .05, Figure 4). The HFD-fed rats treated with pioglitazone for 8 weeks had hepatic CPT-1 protein levels that were approached to those in the RCD-fed group (Figure 4). Administration HFD-fed rats with emodin (80 mg kg^−1^ per day) also significantly upregulated the hepatic protein level of the CPT-1 to 2.4-fold relative to those in vehicle-treated HFD-fed rats (*P* < .05, Figure 4).

## 4. Discussion

Obesity, especially abdominal obesity, has an association with dyslipidemia characterized by increasing TG and decreasing HDL-c concentrations [21]. Compelling evidence, from meta-analysis of a number of clinical studies on a large aggregate of patients, has established an increased level of TG as an independent risk factor for cardiovascular disease [22]. TG is involved in the ectopic accumulation of lipid stores in the liver and is associated with a number of diseases such as metabolic syndrome and type 2 diabetes. High TC levels increase the risk of developing coronary heart disease, and high levels of HDL-c are also a risk factor for coronary heart disease, while high HDL-c is helpful in transporting excess cholesterol to the liver for excretion in the bile [23]. As a result, HDL-c levels are inversely related to this risk [24]. The present study demonstrated that HFD-fed rats showed a significant increase in plasma TC, TG, and LDL-c levels. However, levels of plasma HDL-c in HFD-fed rats were decreased compared with the RCD-group. Similar to pioglitazone treatment, emodin (80 mg kg^−1^ per day) significantly lowered plasma TC, TG and LDL-c levels in rats with HFD-induced obesity. The effect of emodin administration on the atherogenic and coronary artery risk indices is notable.

A large body of evidence indicates that AMPK is involved in the maintenance of lipid and cholesterol homeostasis [10]. Activation of hepatic AMPK (through phosphorylation of its *α*-subunit on Thr172) switches off fatty acid synthesis acutely via increased phosphorylation and inactivation of ACC which reduces the production of malonyl-CoA, an allosteric inhibitor of CPT-1 [25]. CPT-1, regulating acyl-CoA inflow and *β*-oxidation in the mitochondrial outer membrane, is a rate-limiting step for fatty acid oxidation [25]. Inactivation of ACC by AMPK helps promote fatty acid utilization, leading to fat burning in fat and muscle. The study was further to clarify whether the action of emodin on the regulation of lipid disorders is related with AMPK-signaling pathway. The results showed that, concomitant with enhancement of AMPK activity by promoting AMPK phosphorylation, and inhibition of ACC activation by increasing ACC phosphorylation, emodin up-regulated CPT-1 gene expression in hepatocytes, suggesting that emodin may reduce lipid levels via promotion of hepatic fatty acid oxidation mediated by regulating AMPK activation.

Sterol regulatory element binding protein 1 (SREBP-1) is a key lipogenic transcription factor, which directly activates the expression of more than 30 genes (including FAS), dedicated to the synthesis and uptake of fatty acids, cholesterol, and triglyceride [26]. Increased SREBP-1 levels have been found in patients with histologically diagnosed NAFLD [27], and in the fatty livers of obese (ob/ob) mice [28] and obese rats induced by fat-diet feeding [29]. It is known that SREBP-1 is negatively regulated by AMPK [30]. In hepatocytes, it was found that emodin was able to activate AMPK and suppressed the increased gene expression of SREBP-1 and FAS caused by HFD, implying that activation of AMPK stimulated by emodin may lead to prevent SREBP-1 translocation to the nuclei and downregulation of SREBP-1 and FAS expression as well, finally leading to inhibit hepatic lipogenesis. These findings may provide molecular evidence for the use of emodin as a therapy for the management of NAFLD.

**Figure 5:**
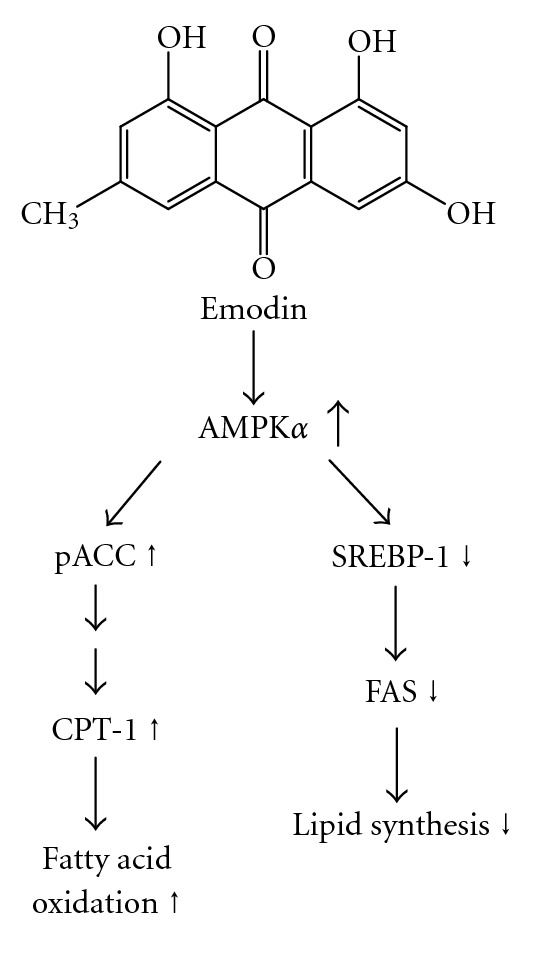
The possible mechanisms of emodin action on the regulation of lipid metabolism in liver of HFD-fed rats.

Emodin is a potent PPAR*γ* agonist that could render it as an attractive therapeutic agent for managing diabetes mellitus which has been documented [9]. The antidiabetic and lipid-modulating effects of emodin involved in the upregulation of L-type calcium channel expression in the pancreas and heart in dyslipidaemic-diabetic rats have also been demonstrated [31]. This study reports, for the first time, emodin could decrease lipid synthesis and increase fatty-acid oxidation through activating AMPK, which further inhibits protein expression in SREBP-1 and lead to the downregulation of FAS, as well as reduction of the transcription activity of ACC and in turn enhancement CPT-1 gene expression (Figure 5). These results together with the previous observations that emodin may possess multi-functional activities against dyslipidemia as well are of importance.

In conclusion, the results of this study showed that emodin enhances CPT-1 expression along with increased AMPK and ACC protein expression and phosphorylation in hepatocytes of HFD-fed rats. The results also indicate that the activation of the AMPK-signaling pathway may play a critical role in the suppression effect of emodin on SREBP-1c and FAS. These findings may provide molecular evidence for the use of emodin as a therapy for the management of hyperlipidaemia and/or fatty liver.

## Figures and Tables

**Table 1:** Changes on the body weight (BW), food, and water intake in HFD-fed rats receiving 8-week treatment with emodin or pioglitazone.

Groups	Initial BW (g rat^−1^)	BW gain (g rat^−1^)	Food intake (g rat^−1^ per day)	Water intake (mL rat^−1^ per day)
RCD-fed				
Vehicle	172.5 ± 7.7	17.8 ± 6.2^b^	19.3 ± 5.9	77.2 ± 8.1
HFD-fed				
Vehicle	173.4 ± 6.9	53.7 ± 7.0	20.3 ± 6.7	86.3 ± 9.4
Emodin (mg kg^−1^ per day)				
40	173.1 ± 8.4	30.7 ± 6.9^a^	21.8 ± 6.9	80.8 ± 9.7
80	172.8 ± 6.7	23.6 ± 5.3^b^	20.3 ± 6.3	78.1 ± 7.9
Pioglitazone (20 mg kg^−1^ per day)	171.6 ± 8.1	20.4 ± 5.8^b^	19.9 ± 7.1	76.7 ± 8.9

Emodin or pioglitazone was dissolved in distilled water for oral administration at the desired doses in a volume of 2 mL kg^−1^ once a day into HFD-fed rats. The vehicle (distilled water) used to dissolve the tested medications was given at the same volume. Values (mean ± SEM) were obtained from each group of 8 animals in each group after 8 weeks of the experimental period. ^a^ *P* < .05 and ^b^ *P* < .01 compared to the values of vehicle-treated HFD-fed rats in each group, respectively.

**Table 2:** Changes in the plasma lipids, atherogenic index (AI), and coronary artery index (CRI) in HFD-fed rats receiving 8-weeks treatment with emodin or pioglitazone.

Groups	Plasma levels (mg dL^−1^)					AI	CRI
	TC	TG	LDL-c	HDL-c	FFAs		
RCD-fed							
Vehicle	72.9 ± 5.8^b^	56.8 ± 6.6^b^	31.8 ± 3.2^b^	46.9 ± 3.4^b^	28.2 ± 2.8^b^	0.7 ± 0.3^b^	1.6 ± 0.4^b^
HFD-fed							
Vehicle	130.2 ± 12.3	128.5 ± 6.3	120.2 ± 4.2	27.8 ± 3.8	61.9 ± 5.3	4.3 ± 0.2	4.7 ± 0.3
Emodin (mg kg^−1^ per day)							
40	99.6 ± 8.3^a^	95.0 ± 5.2^a^	74.3 ± 5.1^b^	39.1 ± 2.9^a^	47.1 ± 3.8^a^	1.9 ± 0.1^b^	2.5 ± 0.3^b^
80	89.1 ± 7.9^a^	80.3 ± 4.8^b^	68.1 ± 4.5^b^	43.9 ± 3.2^b^	38.7 ± 3.2^b^	1.6 ± 0.1^b^	2.0 ± 0.3^b^
Pioglitazone (20 mg kg^−1^ per day)	80.2 ± 8.0^b^	65.6 ± 4.7^b^	48.4 ± 4.3^b^	45.9 ± 4.4^b^	33.2 ± 4.1^b^	1.1 ± 0.2^b^	1.7 ± 0.4^b^

Emodin or pioglitazone was dissolved in distilled water for oral administration at the desired doses in a volume of 2 mL kg^−1^ once a day into HFD-fed rats. The vehicle (distilled water) used to dissolve the tested medications was given at the same volume. Values (mean ± SEM) were obtained from each group of 8 animals in each group after 8 weeks of the experimental period. ^a^ *P* < .05 and ^b^ *P* < .01 compared to the values of vehicle-treated HFD-fed rats in each group, respectively.

## References

[1] Achike FI, To NHP, Wang H, Kwan CY (2011). Obesity, metabolic syndrome, adipocytes and vascular function: a holistic viewpoint. *Clinical and Experimental Pharmacology and Physiology*.

[2] Greenway FL, Caruso MK (2005). Safety of obesity drugs. *Expert Opinion on Drug Safety*.

[3] Zheng CD, Duan YQ, Gao JM, Ruan ZG (2010). Screening for anti-lipase properties of 37 traditional chinese medicinal herbs. *Journal of the Chinese Medical Association*.

[4] Chang CH, Lin CC, Yang JJ, Namba T, Hattori M (1996). Anti-inflammatory effects of emodin from ventilago leiocarpa. *American Journal of Chinese Medicine*.

[5] Huang SS, Yeh SF, Hong CY (1995). Effect of anthraquinone derivatives on lipid peroxidation in rat heart mitochondria: structure-activity relationship. *Journal of Natural Products*.

[6] Iizuka A, Iijima OT, Kondo K (2004). Evaluation of rhubarb using antioxidative activity as an index of pharmacological usefulness. *Journal of Ethnopharmacology*.

[7] Zhou M, Xu H, Pan L, Wen J, Guo Y, Chen K (2008). Emodin promotes atherosclerotic plaque stability in fat-fed apolipoprotein e-deficient mice. *Tohoku Journal of Experimental Medicine*.

[8] Hei ZQ, Huang HQ, Tan HM (2006). Emodin inhibits dietary induced atherosclerosis by antioxidation and regulation of the sphingomyelin pathway in rabbits. *Chinese Medical Journal*.

[9] Xue J, Ding W, Liu Y (2010). Anti-diabetic effects of emodin involved in the activation of ppar*γ* on high-fat diet-fed and low dose of streptozotocin-induced diabetic mice. *Fitoterapia*.

[10] Lage R, Diéguez C, Vidal-Puig A, López M (2008). Ampk: a metabolic gauge regulating whole-body energy homeostasis. *Trends in Molecular Medicine*.

[11] Dong H, Lu FE, Gao ZQ, Xu LJ, Wang KF, Zou X (2005). Effects of emodin on treating murine nonalcoholic fatty liver induced by high caloric laboratory chaw. *World Journal of Gastroenterology*.

[12] Feldeisen SE, Tucker KL (2007). Nutritional strategies in the prevention and treatment of metabolic syndrome. *Applied Physiology, Nutrition and Metabolism*.

[13] Hariri N, Thibault L (2010). High-fat diet-induced obesity in animal models. *Nutrition Research Reviews*.

[14] Van Heek M, Compton DS, France CF (1997). Diet-induced obese mice develop peripheral, but not central, resistance to leptin. *Journal of Clinical Investigation*.

[15] Ding SY, Shen ZF, Chen YT, Sun SJ, Liu Q, Xie MZ (2005). Pioglitazone can ameliorate insulin resistance in low-dose streptozotocin and high sucrose-fat diet induced obese rats. *Acta Pharmacologica Sinica*.

[16] Friedewald WT, Levy RI, Fredrickson DS (1972). Estimation of the concentration of low-density lipoprotein cholesterol in plasma, without use of the preparative ultracentrifuge. *Clinical Chemistry*.

[17] Shanmugasundaram KR, Visvanathan A, Dhandapani K (1986). Effect of high-fat diet on cholesterol distribution in plasma lipoproteins, cholesterol esterifying activity in leucocytes, and erythrocyte membrane components studied: importance of body weight. *The American Journal of Clinical Nutrition*.

[18] Abbott RD, Wilson PWF, Kannel WB, Castelli WP (1988). High density lipoprotein cholesterol, total cholesterol screening, and myocardial infarction. the framingham study. *Arteriosclerosis*.

[19] Folch J, Lees M, Sloane-Stanley GH (1957). A simple method for the isolation and purification of total lipides from animal tissues. *the Journal of Biological Chemistry*.

[20] Brunt EM, Janney CG, Di Bisceglie AM, Neuschwander-Tetri BA, Bacon BR (1999). Nonalcoholic steatohepatitis: a proposal for grading and staging the histological lesions. *American Journal of Gastroenterology*.

[21] Paccaud F, Schlüter-Fasmeyer V, Wietlisbach V, Bovet P (2000). Dyslipidemia and abdominal obesityan assessment in three general populations. *Journal of Clinical Epidemiology*.

[22] Malloy MJ, Kane JP (2001). A risk factor for atherosclerosis: triglyceride-rich lipoproteins. *Advances in Internal Medicine*.

[23] Libby P (2001). Current concepts of the pathogenesis of the acute coronary syndromes. *Circulation*.

[24] Ansell BJ, Watson KE, Fogelman AM, Navab M, Fonarow GC (2005). High-density lipoprotein function: recent advances. *Journal of the American College of Cardiology*.

[25] Saha AK, Ruderman NB (2003). Malonyl-coa and amp-activated protein kinase: an expanding partnership. *Molecular and Cellular Biochemistry*.

[26] Hagen RM, Rodriguez-Cuenca S, Vidal-Puig A (2010). An allostatic control of membrane lipid composition by srebp1. *Febs Letters*.

[27] Kohjima M, Higuchi N, Kato M (2008). Srebp-1c, regulated by the insulin and ampk signaling pathways, plays a role in nonalcoholic fatty liver disease. *International Journal of Molecular Medicine*.

[28] Ahmed MH, Byrne CD (2007). Modulation of sterol regulatory element binding proteins (srebps) as potential treatments for non-alcoholic fatty liver disease (nafld). *Drug Discovery Today*.

[29] Madsen L, Petersen RK, Sørensen MB (2003). Adipocyte differentiation of 3t3-l1 preadipocytes is dependent on lipoxygenase activity during the initial stages of the differentiation process. *Biochemical Journal*.

[30] Li Y, Xu S, Mihaylova MM (2011). Ampk phosphorylates and inhibits srebp activity to attenuate hepatic steatosis and atherosclerosis in diet-induced insulin-resistant mice. *Cell Metabolism*.

[31] Zhao XY, Qiao GF, Li BX (2009). Hypoglycaemic and hypolipidaemic effects of emodin and its effect on l-type calcium channels in dyslipidaemic-diabetic rats. *Clinical and Experimental Pharmacology and Physiology*.

